# Glycochenodeoxycholic acid induces stemness and chemoresistance via the STAT3 signaling pathway in hepatocellular carcinoma cells

**DOI:** 10.18632/aging.103751

**Published:** 2020-08-03

**Authors:** Changying Shi, Jiamei Yang, Longmiao Hu, Boyi Liao, Liang Qiao, Weifeng Shen, Feng Xie, Guoqing Zhu

**Affiliations:** 1 Department of Hepatology, Eastern Hepatobiliary Surgery Hospital, Affiliated to Second Military Medical University, Shanghai, China; 2 National Center for Liver Cancer, Second Military Medical University, Shanghai, China; 3 Department of Biliary Surgery, Eastern Hepatobiliary Surgery Hospital, Affiliated to Second Military Medical University, Shanghai, China; 4 Department of Interventional Radiology, The First Affiliated Hospital of Wenzhou Medical University, Wenzhou, China

**Keywords:** hepatocellular carcinoma, cancer stem cells, glycochenodeoxycholic acid, STAT3 signaling pathway

## Abstract

The poor prognosis of hepatocellular carcinoma (HCC) is primarily attributed to its high frequency of recurrence and resistance to chemotherapy. Epithelial-to-mesenchymal transition (EMT) and the acquisition of cancer stem cells (CSCs) are the fundamental drivers of chemoresistance in HCC. Glycochenodeoxycholic acid (GCDC), a component of bile acid (BA), has been reported to induce necrosis in primary human hepatocytes. In the present work, we investigated the function of GCDC in HCC chemoresistance. We found that GCDC promoted chemoresistance in HCC cells by down-regulating and up-regulating the expression of apoptotic and anti-apoptotic genes, respectively. Furthermore, GCDC induced the EMT phenotype and stemness in HCC cells and activated the STAT3 signaling pathway. These findings reveal that GCDC promotes chemoresistance in HCC by inducing stemness via the STAT3 pathway and could be a potential target in HCC chemotherapy.

## INTRODUCTION

Hepatocellular carcinoma (HCC) is the fifth most common cancer worldwide, with a high mortality rate due to poor prognosis. According to a study, approximately 50% of newly diagnosed cases of HCC occur in China [1]. The treatment mainly involves surgery, radiotherapy, and chemotherapy; however, these are often ineffective.

The poor prognosis of HCC is attributed to the high frequency of recurrence and resistance to chemotherapy. The presence of cancer stem cells (CSCs), a distinct subpopulation of cells with high self-renewal ability or stemness, contributes to poor prognosis and high mortality of HCC [2]. Furthermore, this stemness feature confers resistance to existing drugs[3]. Enhanced expression of several CSC-related markers, such as epithelial cell adhesion molecule (EpCAM), CD44, CD133, and Nanog, is associated with poor prognosis of HCC [4–7]. Similarly, alterations in stemness-related signaling pathways, such as Wnt/β-catenin, Notch, and Hedgehog pathways, resulted in chemoresistance and recurrence of HCC [8–10]. Therefore, CSCs and their associated pathways are becoming the focus of potential therapies for HCC.

The occurrence of HCC is closely related to chronic liver injury. The obstruction of bile duct and accumulation of bile acid (BA) can damage the liver [11]. Moreover, dysregulation of BA metabolism is associated with the development of HCC [12]. Glycochenodeoxycholic acid (GCDC), a component of BA, induced hepatocyte necrosis in patients with obstructive cholestasis [13]. Recently, a study indicated GCDC could induce autophagy and metastasis in HCC cells [14]. Here, we demonstrated that GCDC promotes chemoresistance of HCC cells by inducing stemness via the STAT3 signaling pathway.

## RESULTS

### GCDC enhances chemoresistance of HCC cells

**Figure 1:**
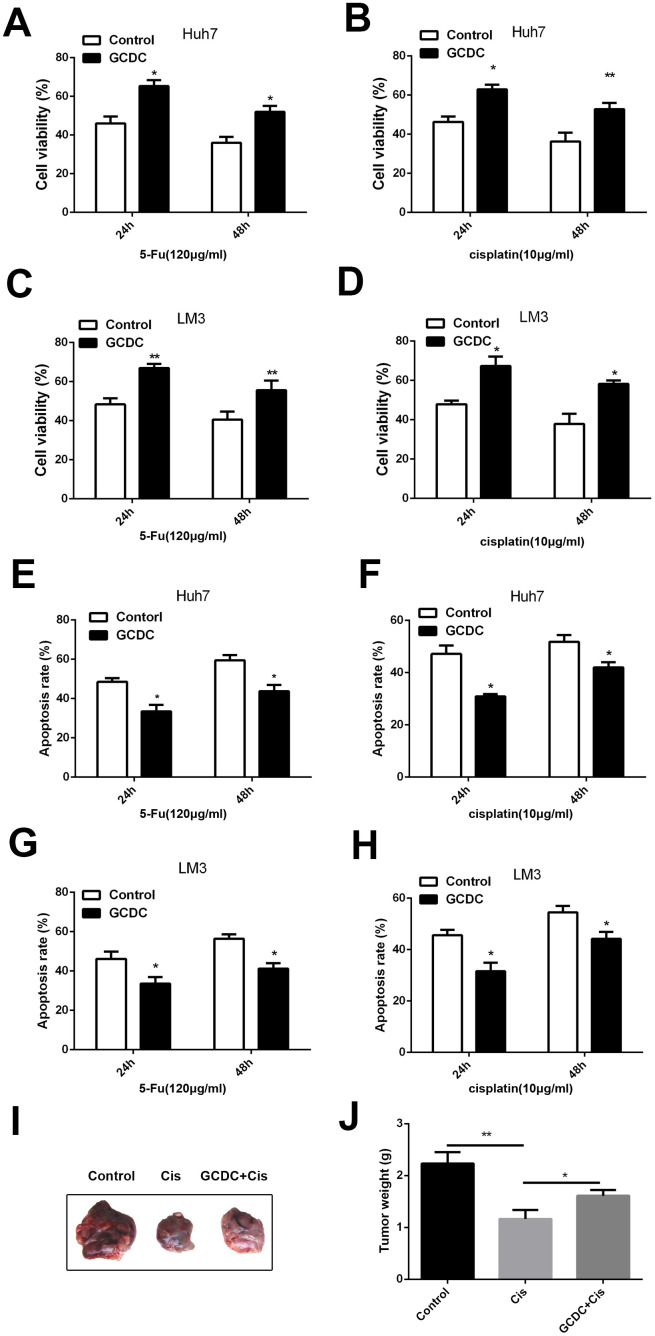
**GCDC enhances chemoresistance in HCC cells**. (**A–D**) Cell viability was detected using the CCK-8 assay. *P<0.05, **P<0.01. (**E–H**) Cell apoptosis was analyzed by flow cytometry. *P<0.05. (**I–J**) GCDC enhanced the ability of chemoresistance of HCC cells in vivo. Huh7 cells (5×10^6^) were pretreated with GCDC and then were implanted in the right subcutaneous armpit area of nude mice. Then the cisplatin (4mg/kg) were injected in tumor every 3 days. After 27 days, the mice were sacrificed and the weight of the tumor was measured.*P<0.05, **P<0.01.

We first determined the effect of GCDC on chemoresistance of Huh7 and LM3HCCcell lines. The cells were treated with GCDC (200μm) and chemotherapeuticdrugs5-fluorouracil (5-FU) (120μg/mL) and cisplatin (10 μg/mL), and the cell viability was detected using the CCK-8 assay. Figure 1A, 1B show that GCDC increased the cell viability of almost 20% in Huh7cells treated with5-FU and cisplatin. Similar results were obtained for LM3 cell line (Figure 1C, 1D). Next, we studied the effect of GCDC on drug-induced apoptosis. As shown in Figure 1E, 1F, compared with the chemotherapeutic drugs treated alone group, the GCDC-treated group showed reduced apoptosis in both Huh7 and LM3 cell lines after treatment with 5-FU and cisplatin. These results demonstrated that GCDC promoted chemoresistance in HCC cells.

**Figure 2:**
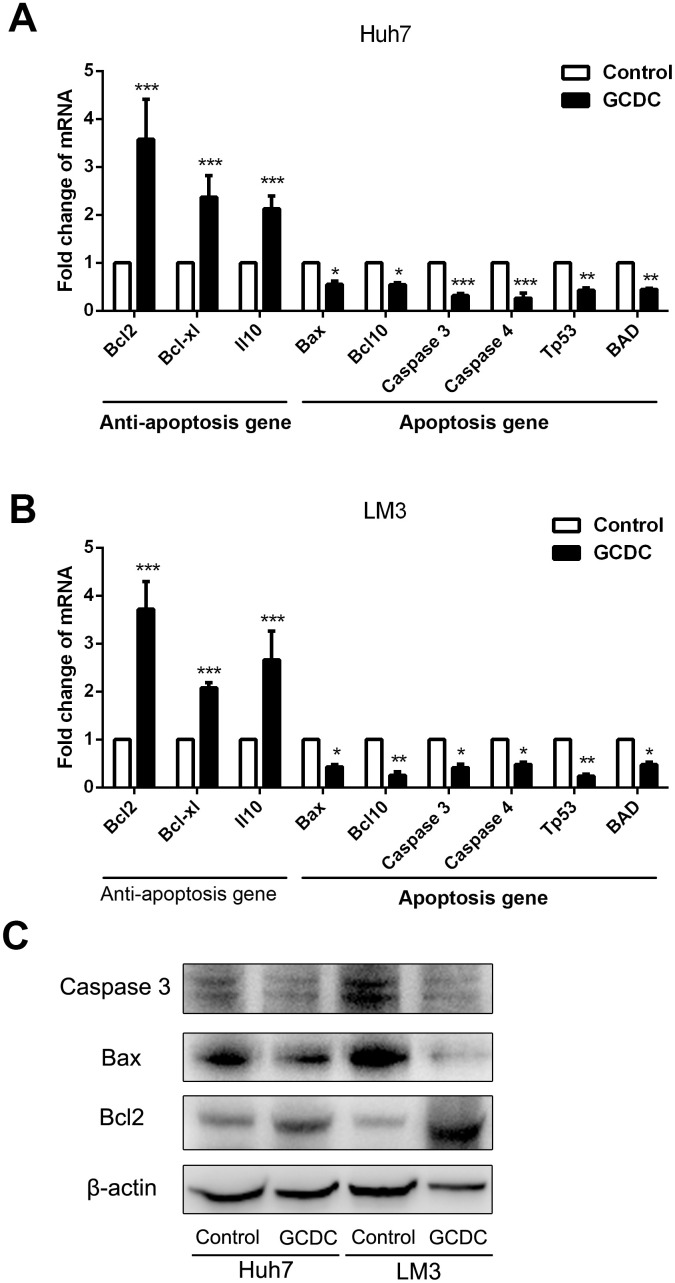
**GCDC regulates the expression of apoptotic and anti-apoptotic genes.** (**A**, **B**) The expression of apoptotic and anti-apoptotic genes in HCC cells was examined by reverse transcriptase-polymerase chain reaction (RT-PCR) assays. *P<0.05, **P<0.01, ***P<0.001. (**C**) The expression of apoptotic and anti-apoptotic genes in HCC cells was examined by Western blot.

We further examined the effect of GCDC on the expression of anti-apoptotic (Bcl2, Bcl-xl and Il10) and apoptotic genes (Bcl10, Caspase 3, Caspase 4, Tp53, BAD) using RT-PCR assays and Western blot. As shown in Figure 2, compared with the control group, the expression of apoptotic genes was suppressed, whereas that of anti-apoptotic genes increased in the GCDC-treated group. These results showed that GCDC promoted cell viability in 5-FU- and cisplatin-treated HCC cells.

### GCDC induces EMT and stemness in HCC cells

**Figure 3:**
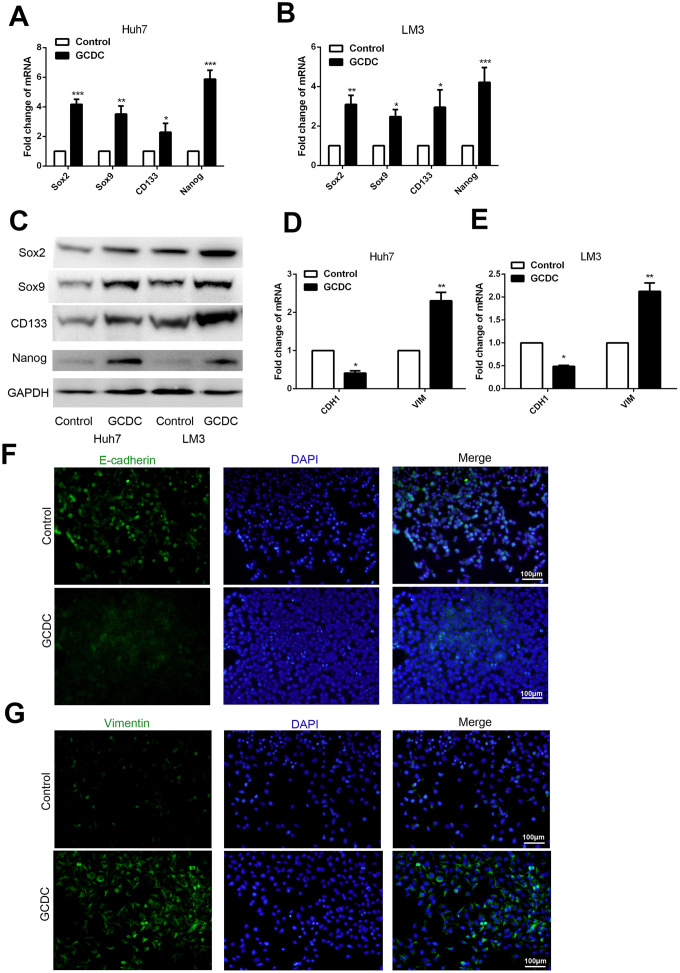
**GCDC promotes EMT and stemness in HCC cells**. (**A, B**) Reverse-transcriptase polymerase chain reaction (RT-PCR) was used to detect the expression of stem cell markers (Sox2, Sox9, Nanog, and CD133) in HCC cells. *P<0.05, **P<0.01, ***P<0.001. (**C**) Western blotting was used to detect the expression of stem cell markers (Sox2, Sox9, Nanog, and CD133) in HCC cells. (**D**, **E**) RT-PCR was performed to examine the expression of E-cadherin and vimentin in HCC cells. *P<0.05, **P<0.01. (**F**, **G**) The protein expression of E-cadherin and vimentin was confirmed by immunofluorescence.

The presence of CSCs in several cancers has been shown to confer chemoresistance. Therefore, we studied this property in HCC cells following treatment with GCDC. RT-PCR and western blotting were used to detect the expression of stem cell markers (Sox2, Sox9, Nanog, and CD133). The results showed that GCDC promoted the expression of Sox2, Sox9, Nanog and CD133 at both mRNA and protein levels (Figure 3A–3C). As epithelial–mesenchymal transition (EMT) promotes the acquisition of CSC phenotype and development of chemoresistance, we studied the expression of epithelial cell marker (E-cadherin) and mesenchymal cell marker (vimentin) by RT-PCR. As shown in Figure 3D–3G, compared with the control group, the mRNA and protein level of E-cadherin was down-regulated, and that of vimentin was up-regulated in GCDC-treated Huh7and LM3 cells. These results suggested that GCDC promoted chemoresistance in HCC cells by conferring EMT phenotype and CSC properties.

### GCDC activates the STAT3 signaling pathway in HCC cells

**Figure 4:**
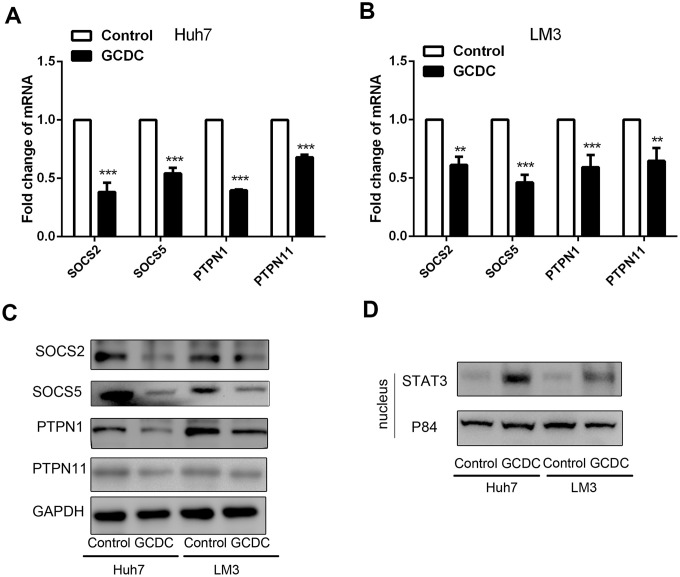
**GCDC activates the STAT3 signaling pathway in HCC cells**. (**A, B**) The mRNA expression of SOCS2, SOCS5, PTPN1, and PTPN11 was detected by reverse-transcriptase polymerase chain reaction (RT-PCR). **P<0.01, ***P<0.001. (**C**) Western blotting was used to detect the expression of SOCS2, SOCS5, PTPN1, and PTPN11. (**D**) The expression of STAT3 in the cell nucleus was examined by western blotting. P84 was used as an internal reference.

The STAT3 signaling pathway is known to be involved in cell survival and proliferation. To further investigate its role in GCDC-induced chemoresistance, we studied whether GCDC activated theSTAT3 pathway. We first examined the expression of several negative regulators of STAT3 signaling, including suppressor of cytokine signaling (SOCS) members, SOCS2 and SOCS5, and tyrosine phosphatases, PTPN1 and PTPN11, in GCDC-treated HCC cells. As shown in Figure 4A–4C, the expression of all four negative regulators was down-regulated in GCDC-treated Huh7and LM3 cells. Next, the expression of STAT3 in the nucleus (activated STAT3) was detected. These results implied that GCDC promoted the activation of the STAT3 pathway in both Huh7 and LM3 cells (Figure 4D).

### GCDC promotes chemoresistance of HCC through activation of the STAT3 signaling pathway

**Figure 5:**
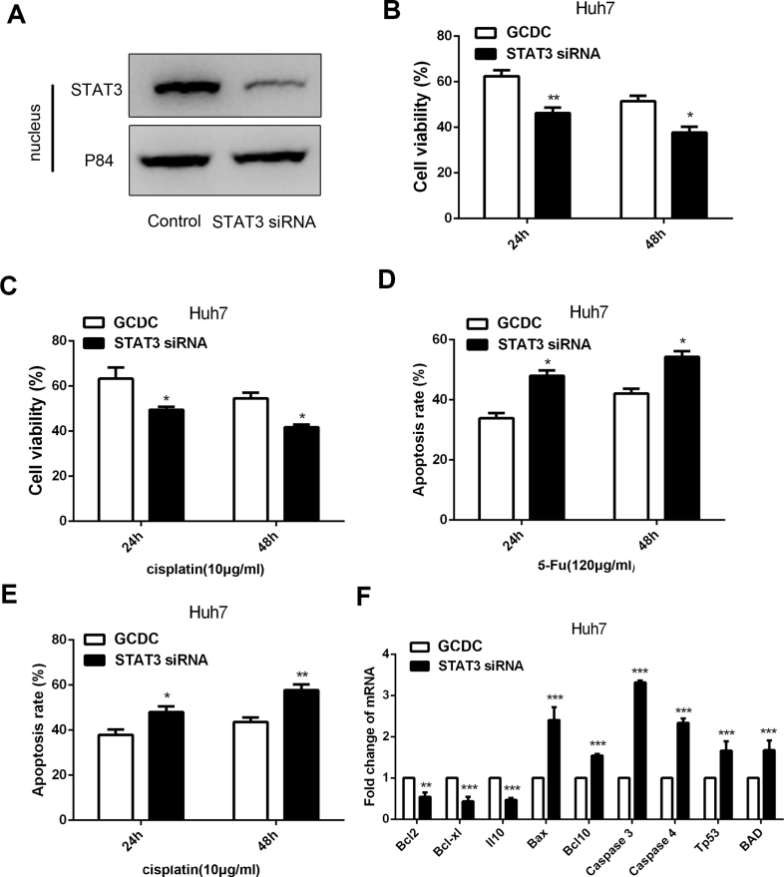
**GCDC promotes chemoresistance of HCC through the STAT3 signaling pathway.** (**A**) Western blotting was used to assess the effect of siRNA on the STAT3 expression. P84 was used as an internal reference. (**B**, **C**) Cell viability was detected by the CCK-8 assay. *P<0.05, **P<0.01. (**D**, **E**) Cell apoptosis was analyzed using flow cytometry. *P<0.05. (**F**) The expression of apoptotic and anti-apoptotic genes in Huh7 cells was examined by reverse-transcriptase-polymerase chain reaction (RT-PCR) assays. **P<0.01, ***P<0.001.

To further demonstrate that the STAT3 signaling pathway was involved in GCDC-induced chemoresistance in HCC cells, *STAT3*siRNA was used to down-regulate its expression in HCC cells. Western blotting confirmed that the siRNA efficiently knocked down the expression of *STAT3* in the nucleus of Huh7cells (Figure 5A). We further examined the chemoresistance in GCDC-treated HCC cells when *STAT3* was suppressed. As expected, the decrease in *STAT3* reversed the GCDC-induced chemoresistance (Figure 5B–5E). Further, the expression of apoptotic genes increased and that of anti-apoptotic genes decreased in GCDC-treated Huh7cells when *STAT3* was suppressed (Figure 5F). These data indicated that the activation of the STAT3 signaling pathway promoted GCDC-induced chemoresistance in HCC cells.

## DISCUSSION

The high mortality rate of HCC is mainly attributed to its extreme resistance to systemic chemotherapy. We demonstrated that GCDC promoted chemoresistance of HCC cells *in vitro* by down-regulating the expression of apoptotic genes and up-regulating the expression of anti-apoptotic genes. Furthermore, GCDC induced stemness in HCC cells via EMT and activation of the STAT3 signaling pathway. These results proved that GCDC contributes to the chemoresistance of HCC and could be a potential target in HCC therapy.

The EMT phenotype and the presence of CSCs are the major contributing factors to chemoresistance in several kinds of cancers, such as pancreatic cancer, ovarian cancer, colorectal cancer, and HCC [15–18]. In the present work, the expression of CSC markers, such as Sox2, Sox9, Nanog, and CD133, was shown to be up-regulated in GCDC-treated HCC cells. The expression of Nanog and CD133has been reported to correlate with poor clinical outcome in patients with HCC [19, 20]. Similarly, Sox2 and Sox9 contribute to HCC progression and malignancy [21, 22]. EMT involves the loss of epithelial characteristics due to down-regulation of E-cadherin and acquisition of mesenchymal properties by up-regulation of the mesenchymal protein vimentin. The decrease in the expression of E-cadherin and an increase in the expression of vimentin in HCC cells following treatment with GCDC suggested that stemness and EMT phenotype contributed to GCDC-induced chemoresistance in HCC cells.

Studies have demonstrated that the JAK/STAT3 signaling pathway contributes to cell survival and chemotherapeutic resistance in cancers [23, 24]. Furthermore, it enhances the development of CSC-like characteristics [25]. For example, the CSC marker, Nanog, is induced by the STAT3pathway in liver tumor-initiating cells [26]. Similarly, members of the SOCS and PTPN families have been demonstrated to negatively affect the JAK/STAT signaling pathway. We found that GCDC activated the STAT3 signaling pathway by repressing the expression of several negative regulators of STAT3 signaling, including SOCS2, SOCS5, PTPN1, and PTPN11 in HCC cells. GCDC-induced resistance to drugs was inhibited when the expression of *STAT3* was suppressed by siRNA in HCC cells. These results demonstrated that the STAT3 signaling pathway is involved in GCDC-induced chemoresistance of HCC cells. To summarize, our results showed that the treatment with GCDC enhanced the chemoresistance of HCC cells by inducing CSC-like characteristics and EMT phenotype, and activating the STAT3 signaling pathway via suppression of the expression of SOCS2, SOCS5, PTPN1, and PTPN1. Therefore, GCDC could serve as a potential target for the prognosis and therapy of HCC.

## MATERIALS AND METHODS

### Cell culture and reagents

HCC cell lines, Huh7 and LM3, were cultured in Dulbecco’s modified Eagle’s medium (high glucose) (GIBCO, Invitrogen, US) with 10% fetal bovine serum (FBS) and 1% penicillin/streptomycin. The cells were incubated at 37°Cin a humidified atmosphere containing 5% CO_2_.

### Cell proliferation and cytotoxicity assay

Chemotherapy-induced cell death was determined by cell counting kit-8 assay [27]. Huh7 and LM3 cells were seeded in a 96-well plate at a density of 8 × 10^3^ cells/well and incubated with GCDC and treated with chemotherapeutic drugs (5-FU and cisplatin) for 24 h and 48 h, respectively. Next, the cells were washed with phosphate-buffered saline (PBS), and cell counting kit-8 (CCK-8) solution (1/10 the volume of media) was added for 1 h. The cell viability was detected at 450 nm using a microplate reader.

### Apoptosis assay

A total of 1 × 10^5^ cells were seeded in a 6-well plate and treated with GCDC and chemotherapeutic drugs for 24 h and 48 h, respectively. Next, the cells were washed with PBS and resuspended in the PBS and stained with Annexin V and propidium iodide (PI) according to manufacturer’s instructions (BD Biosciences; San Diego, CA, USA). Flow cytometry was used to analyze the proportion of apoptotic cells.

### Reverse transcriptase-polymerase chain reaction (RT-PCR)

Total RNA was isolated using the TRIzol reagent (Invitrogen; Carlsbad, CA, USA) according to the manufacturer’s instructions. Next, cDNA was synthesized using the Prime Script RT reagent Kit (Takara; Kyoto, Japan). Reverse transcriptase-polymerase chain reaction (RT-PCR) was performed using the SYBR Green PCR Kit (Applied Biosystems, US) according to the manufacturer’s instructions.

### Western blotting

Proteins were resolved using 20% sodium dodecyl sulfate-polyacrylamide gel electrophoresis (SDS-PAGE), followed by transfer of separated proteins onto a nitrocellulose membrane. Non-specific antigens on the membrane were blocked by incubating the membrane in 1×TBST (Tris-buffered saline with 0.1% Tween-20) containing 5% non-fat skim milk at room temperature for 1 h. Afterward, the membrane was incubated overnight with primary antibodies at 4°C followed by incubation with the secondary antibody (goat anti-mouse or anti-rabbit IgG antibody) at room temperature for 1 h. The primary antibodies used was anti-caspase3 (Abcam, US, 1;500), anti-Bax (R&D system, US, 1:1000), anti-Bcl2 (Abcam, US, 1;500), anti-Sox2 (Abcam, US, 1:1000), anti-Sox9 (Abcam, US, 1:1000), anti-CD133 (Proteintech, US, 1;1000), anti-Nanog (Proteintech, US, 1:1000), anti-SOCS2 (Abcam, US, 1:1000), anti-SOCS5 (Abcam, US, 1:500), anti-PTPN1 (Proteintech, US, 1:2000), anti-PTPN11 (Proteintech, US, 1:500), anti-STAT3 (Proteintech, US, 1:1000) The immunoblots were developed using the BeyoECL kit (Beyotime, China) and Tanon 5200 system (Tanon, China).

### Immunofluorescence

Cells were seeded in a 48-well plate and fixed with 4% paraformaldehyde for 15 min, followed by treatment with 0.1% Triton X-100 for 10 min. The samples were blocked with 3% bovine serum albumin (BSA) at 37°C for 30 min and incubated with primary antibodies against E-cadherin (Abcam, US, 1:200) and vimentin (Abcam, US, 1:200) overnight at 4°C. Subsequently, the cells were incubated with conjugated secondary antibodies at 37°C for 30 min. Nuclei were stained with DAPI for 2 min, and samples were observed under a microscope (Olympus ZX71; Olympus Corp., Japan).

### Short interfering RNA interference

A short interfering RNA (siRNA) sequence (GAGAAGCAUCGUGAGUGA[dT] [dT]) targeting STAT3 was designed by OBiO Technology (Shanghai, China). A scramble siRNA sequence was used as a negative control. Western blotting was used to detect the interference efficiency of siRNA.

### Animal model

Nude mice (6 weeks old) were purchased from Shanghai Experimental Animal Center, Chinese Academy of Science. HCC cells in different groups were implanted in the right subcutaneous armpit area of nude mice. The mice were sacrificed at the end of the experiment and the weight of the tumor was measured. All procedures involving animals were performed in accordance with the institutional animal welfare guidelines of Second Military Medical University.

### Statistical analysis

The data were analyzed using GraphPad Prism 6.0 (GraphPad Software). Quantitative data are expressed as mean ± standard deviation (SD) for each experiment. Significance between the groups was determined using Student’s *t*-test. For all analyses, P < 0.05 was considered significant.
